# HCV RNA Activates APCs via TLR7/TLR8 While Virus Selectively Stimulates Macrophages Without Inducing Antiviral Responses

**DOI:** 10.1038/srep29447

**Published:** 2016-07-07

**Authors:** Yuwei Zhang, Mohamed El-Far, Franck P. Dupuy, Mohamed S. Abdel-Hakeem, Zhong He, Francesco Andrea Procopio, Yu Shi, Elias K. Haddad, Petronela Ancuta, Rafick-Pierre Sekaly, Elias A. Said

**Affiliations:** 1 Centre de recherche du centre Hospitalier de l’Université de Montréal (CRCHUM), Hôpital Saint-Luc, Québec H2X 0A9, Canada; 2 Département de Microbiologie, Infectiologie et Immunologie, Faculté de Médecine, Université de Montréal, Montréal, Québec H3T 1J4, Canada; 3 Vaccine and Gene Therapy Institute-Florida (VGTI-FL), Port Saint Lucie, Florida 3498, USA; 4 Research Institute of the McGill University Health Centre, Montreal, Quebec, Canada; 5 Department of Microbiology and Immunology, Faculty of Pharmacy, Cairo University, Kasr El-Aini, Cairo 11562, Egypt; 6 Case Western Reserve University, Cleveland, Ohio, USA; 7 Department of Microbiology and Immunology, College of Medicine and Health Sciences, Sultan Qaboos University, Muscat, the Sultanate of Oman

## Abstract

The innate and adaptive immune systems fail to control HCV infection in the majority of infected individuals. HCV is an ssRNA virus, which suggests a role for Toll-like receptors (TLRs) 7 and 8 in initiating the anti-viral response. Here we demonstrate that HCV genomic RNA harbours specific sequences that initiate an anti-HCV immune response through TLR7 and TLR8 in various antigen presenting cells. Conversely, HCV particles are detected by macrophages, but not by monocytes and DCs, through a TLR7/8 dependent mechanism; this leads to chloroquine sensitive production of pro-inflammatory cytokines including IL-1β, while the antiviral type I Interferon response is not triggered in these cells. Antibodies to DC-SIGN, a c-type lectin selectively expressed by macrophages but not pDCs or mDCs, block the production of cytokines. Novel anti-HCV vaccination strategies should target the induction of TLR7/8 stimulation in APCs in order to establish potent immune responses against HCV.

Infection with Hepatitis C virus (HCV) affects 185 million people worldwide, which makes it one of the main public health problems^1^,^2^. HCV infection can result in chronic hepatitis with increased risk of progression to cirrhosis and hepatocellular carcinoma (HCC)^3^. Adaptive immunity in HCV infection is usually delayed, regardless of the outcome of the disease progression, which suggests a lack of proper innate immune responses^4^,^5^. This is likely due to the capacity of HCV to evade detection by the innate immune cells^4^. Different pattern recognition receptors (PRRs) have the capacity to recognize pathogen associated molecular patterns (PAMPs) in HCV and therefore to trigger antiviral and pro-inflammatory innate immune responses^6^. For this stimulation to take place, HCV has to be recognized by endosomal sensors particularly TLR3, 7 and 8 to detect viral RNA^7^,^8^,^9^,^10^. Furthermore, the recognition of HCV genomic RNA by the retinoic acid inducible gene-I (RIG-I)^11^,^12^,^13^ upon uncoating of the virus and genomic amplification during HCV infection, can also initiate anti-viral responses. Although poorly understood, the role of TLR3 in the detection of HCV extracellular double stranded RNA (dsRNA) replicative intermediates was reported^8^,^14^. TLR7 and TLR8 can detect single stranded RNA (ssRNA) molecules. The potential implication of TLR7 in the innate immune response against HCV was postulated^4^,^15^. In this regard, pDCs have been shown to respond to TLR7-ligation *in vitro* using Huh-7 infected cells^16^. Moreover, the presence of a GU-rich sequences in the HCV genome was shown to be detected by TLR7^17^. Furthermore, single nucleotide polymorphisms (SNPs) in TLR7 and TLR8 were shown to be associated with a decrease in the magnitude of inflammation and fibrosis in male patients with chronic HCV-infection, and with the response to IFN-α−based therapy as well as the susceptibility to HCV infection^18^,^19^,^20^. However, the underlying mechanisms for the role of TLR-7 in HCV infection are not fully understood. Moreover, little is known about the role of TLR8 in the innate immune responses against HCV. Here we dissect the roles of TLR7 and TLR8 in the detection of specific motifs in the HCV genomic RNA and the differential stimulation of mDCs, pDCs, macrophages and monocytes by HCV particles.

## Results

### HCV genomic RNA encodes GU-rich sequences that stimulate TLR7/TLR8

**Figure 1:**
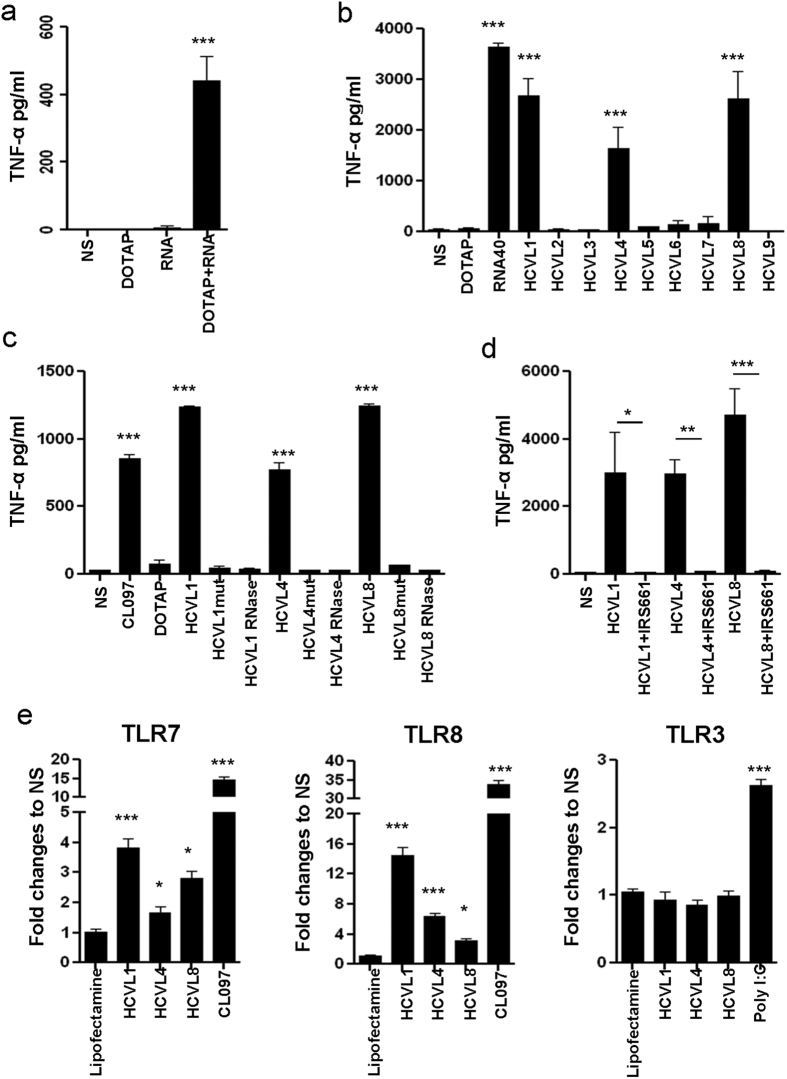
Identification of three novel immunogenic GU-rich sequences in the HCV genome that specifically trigger TLR7 and TLR8 signaling. (**a**) PBMCs from healthy donors (10^6^ cells/ml) were stimulated for 24 h with HCV-RNA extracted from viral particles (RNA), DOTAP alone, or HCV-RNA (10^6^ copies) fused with DOTAP (DOTAP + RNA). Unstimulated cells (NS) were used as controls. (**b**) Shown is a screening for the immunogenic potential of GU-rich single stranded RNA (ssRNA) sequences identified in the HCV genome. Briefly, PBMCs (10^6^ cells/ml) were stimulated for 24 h with different DOTAP-fused ssRNAs (7.5 μg/ml). DOTAP-fused RNA-40 (7.5 μg/ml) was used as a positive control. DOTAP alone and NS cells were used as negative controls. (**c**) PBMCs were stimulated with the original ssRNAs sequences and with sequences in which U to A mutations or RNase-treated ssRNA were used. (**d**) PBMCs were stimulated with the indicated immunogenic ssRNAs sequences in the presence of absence of the TLR7/8 inhibitor IRS661 (10 μg/ml). (**a–d**) Levels of TNF-α in cell culture supernatants were quantified by ELISA. Results represent mean ± SD values generated with PBMCs from 3 different individuals. (**e**) 293 T cells expressing TLR7, TLR8 or TLR3 were transfected with the pNfity2-luc vector, which includes the luciferase gene under the control of NF-κB. Cells were stimulated with selected immunogenic HCV ssRNA sequences (HCVL1, 4 and 8) fused with lipofectamine2000 and the luciferase activity was measured in cell lysates. Results are expressed as ratio of luciferase activity observed with cells exposed to HCV-RNA sequences to that with cells exposed to lipofectamine alone. CL097 and Poly-IC were used as typical ligands for TLR7/8 and TLR3 triggering, respectively. Values represent mean ± SD of triplicate values generated with cells in one experiment representative of results obtained in three different experiments. Dunnett’s test *p*-values relative to DOTAP or lipofectamine are indicated on the graphs (*p < 0.05, **p < 0.01, ***p < 0.001).

To determine if HCV genome can trigger both TLR7 and TLR8, PBMCs, isolated from healthy donors, were incubated for 24 hours with HCV RNA isolated from viral particles; TNF-α production was measured by ELISA. Our results (Fig. 1a) demonstrated that HCV RNA significantly induced TNF-α production following 24 h of stimulation (439 pg/ml, *p* < 0.0001) compared to control non-stimulated cells. To identify the specific RNA sequences involved in the stimulation of TNF production, we screened the HCV genome and identified 9 new GU-rich sequences (HCVL 1–9) that could potentially trigger TLR7/8 (Supplementary Table S1). Interestingly, three out of these nine sequences (HCVL1, 4 and 8) were able to trigger TNF-α production by PBMCs at levels comparable to those generated upon stimulation with the reference sequence RNA40^21^ (2,300, 1,800, 2,100 and 3,400 pg/ml for HCVL1, 4, 8 and RNA40 respectively, *p* < 0.0001; Fig. 1b). The specificity of stimulation of these sequences was further assessed by introducing mutations (G or U to A) or by treatment with RNase. Both site-directed mutations and RNase treatment completely abrogated the capacity of these sequences to trigger TNF-α production by PBMCs (*p* < 0.0001; Fig. 1c).

To demonstrate that TLR7/8 triggering was upstream of HCV RNA-mediated stimulation of these cells, we used IRS661, a specific inhibitor of these TLRs^22^. The capacity of HCVL1, 4 and 8 to stimulate TNF-α production by PBMCs was significantly inhibited when the stimulation was carried out in the presence of IRS661 (Fig. 1d, *p* < 0.0001). To confirm the role of TLR7 and TLR8 in detecting the HCV-encoded sequences, we used HEK cells stably expressing TLR7 or TLR8. These cells were transiently transfected with a plasmid bearing the luciferase-encoding gene under the control of NF-κB-dependent promoter to monitor TLR activation. These cells were exposed to either HCV-encoded sequences or to the CL097 ligand (as a positive control). A significant increase in luciferase activity was observed when cells were exposed to HCV sequences (4-, 2- and 3- fold increase for HCVL-1, 4 and 8, respectively in TLR7 expressing cells, *p* < 0.0001, and 15-, 6- and 3- fold increase, respectively in TLR8 expressing cells, (*p* < 0.0001, Fig. 1e). In contrast, the HCV-encoded sequences were not able to induce any luciferase activity under the same conditions in HEK cells expressing TLR3 alone, which detects dsRNA, while Poly I:C was able to induce luciferase expression in these cells (2.8- fold increase; Fig. 1e). Together, our results showed that the GU-rich sequences, identified herein within the HCV genome, have the capacity to significantly induce cell activation in a TLR7 and TLR8-specifc fashion.

### HCV-encoded TLR7/8 ligands induce cytokines and chemokines production by APCs and DC maturation

We further assessed the capacity of these three RNA sequences to induce the production of a panel of cytokines and chemokines by different APCs including isolated monocytes, myeloid DCs (mDCs) and plasmacytoid DCs (pDCs) (>98% purified by enrichment). Stimulation of these cells using HCVL1, 4 and 8 induced the production of TNF-α, IL-6, IL-1β, IL-10, IL-12p70, CXCL9 and CXCL10 by these cells to levels comparable to those induced by the chemical ligand of TLR7/8, CL097, as monitored by Cytometric Bead Array (CBA; Table 1). Of note, introduction of G or U to A mutations by site-directed mutagenesis in these sequences also diminished their capacity to stimulate APCs to produce all measured cytokines and chemokines (Table 1). However, the profile of cytokine production was different in monocytes, mDCs, and pDCs. Although, high levels of TNF-α and IL-6 were induced in monocytes, mDC, and pDC upon stimulation, CXCL9 and CXCL10 were mainly produced by pDCs, whereas IL-1β, IL-12p70, and IL-10 were produced by monocytes and mDCs but barely detected in supernatants of stimulated pDCs (Table 1).

**Figure 2:**
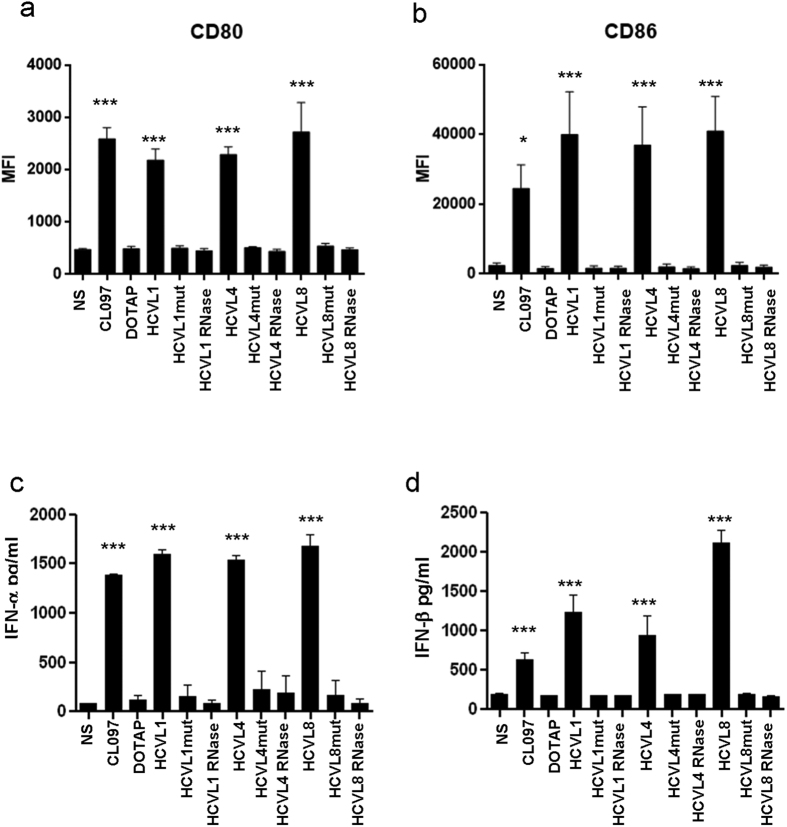
Maturation and type I IFN induction by pDCs. pDCs were isolated by negative selection using magnetic beads and stimulated (10^5^ cells/200 μl) for 24 h with indicated HCV ssRNAs sequences (7.5 μg/ml). In addition to native HCV ssRNA sequences, pDC were exposed to ssRNAs sequences with U to A mutations or RNase-treated ssRNA. **(a,b)** Shown are results of flow cytometry analysis of CD80 and CD86 expression on viable (Vivid-) pDCs cultured under the indicated experimental conditions. **(c,d)** Levels of IFN-α and IFN-β were quantified in cell culture supernatants by ELISA. Results represent mean ± SD values generated with pDCs isolated from 3 different individuals. Dunnett’s test *p*-values relative to NS cells are indicated on the graphs. (*p < 0.05, **p < 0.01, ***p < 0.001).

Incubation of pDCs, freshly isolated from blood, with HCVL1, 4 and 8 led to the maturation of pDCs as observed by the up-regulation of the surface expression of CD80 (MFI = 2,161, 2,267, 2,711 vs control 476, *p* < 0.0001, Fig. 2a) and CD86 (MFI = 43,233, 39709, 44,739 vs control 2,739, *p* < 0.0001; Fig. 2b). Moreover, this stimulation induced the production of IFN-α (1,600, 1,500 and 1,700 pg/ml respectively, *p* < 0.0001) and IFN-β (1,300, 1,000 and 2,250 pg/ml respectively, *p* < 0.0001) by pDCs (Fig. 2c,d respectively).

### Stimulation of monocytes and pDCs but not macrophages and mDCs with HCV-encoded GU-rich sequences inhibits hepatocyte infection with HCV in a type I IFN-dependent manner

**Figure 3:**
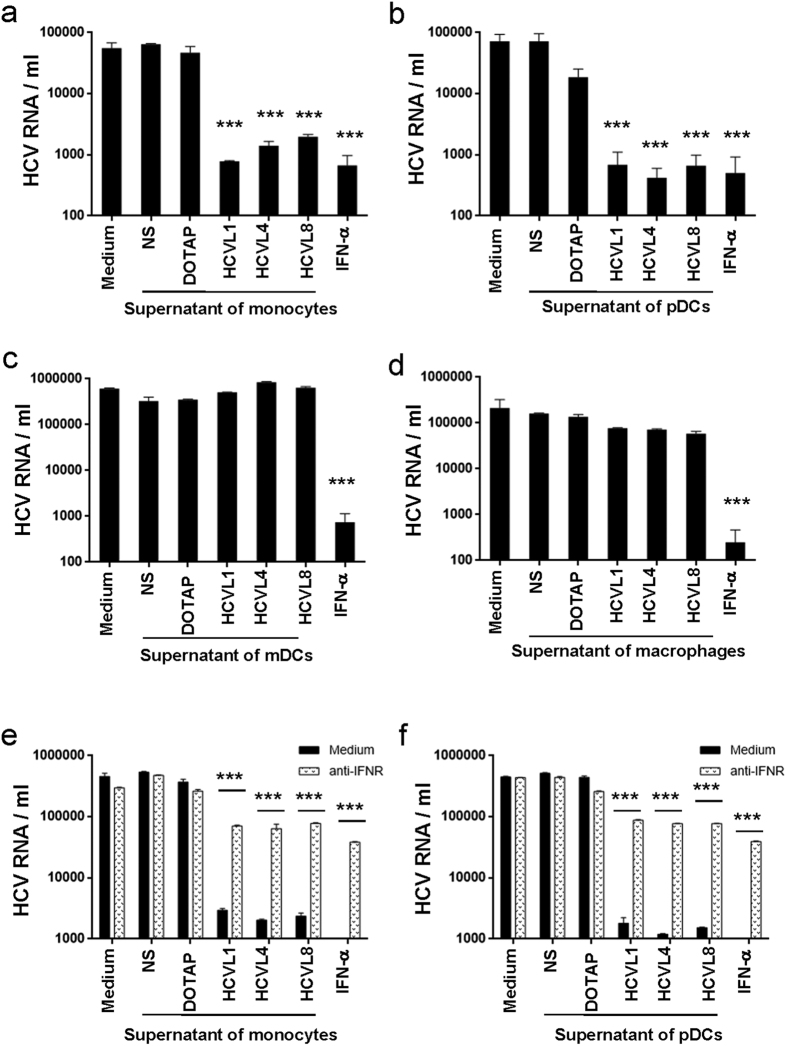
Supernatants from Monocytes and pDCs but not mDC and macrophages exposed to HCV ssRNA inhibit HCV replication in an IFN-dependent manner. (**a–d**) Peripheral blood monocytes (**a**), pDCs (**b**), and mDCs (**c**), were isolated from 3 healthy donors by negative selection using magnetic beads. Macrophages were generated by culturing monocytes in X-vivo 15 medium for 6 days in the presence of GM-CSF (50 ng/ml) (**d**). Cells (10^5^ cells/200 μl) were stimulated with HCV ssRNA sequences (7.5 μg/ml) and cell-free supernatants were harvested at 24 h post-stimulation. In parallel, Huh7.5 cells (2 × 10^4^/200 μl) were incubated with HCV (2 × 10^5^ copies) for 4 h, then washed and cultured in DMEM in the presence or absence of cell culture supernatants (1/10) or IFN-α (100 U/ml). At day 3 post-infection, HCV RNA was extracted from the supernatants and quantified by real-time RT-PCR. (**e,f**) HCV-infected Huh7.5 cells (2 × 10^4^/200 μl) were incubated in the presence or absence of an IFN receptor blocking antibody (10 μg/ml) for 1 h, and then supernatants from HCV ssRNA-exposed monocytes (**e**) or pDCs (**f**) were added to the cells. Levels of HCV RNA were quantified in cell culture supernatants by real-time RT-PCR. Results represent mean ± SD values generated with cells from 3 different individuals. Dunnett’s test *p*-values calculated relative to NS cells are indicated on the graphs. (*p < 0.05, **p < 0.01, ***p < 0.001).

We next investigated the potential anti-viral activity of cytokines produced by DCs upon stimulation with the HCV-encoded sequences identified in this study. Considering the infiltration of macrophages into the liver of HCV-infected subjects and their contribution to chronicity^10^, studies were performed in parallel on monocyte-derived macrophages. We infected Huh7.5 cells with HCV in the presence or absence of supernatants from monocytes, pDC, mDCs or macrophages stimulated or not with HCVL1, 4 or 8. Our results showed that the presence of supernatants from monocytes or pDCs stimulated with HCVL1, 4 or 8 reduced HCV replication in Huh7.5 cells by 2 Log_10_ (*p* < 0.0001), in a way similar to the impact of IFN-α on HCV replication (Fig. 3a,b). In contrast, supernatants from mDCs and macrophages stimulated with HCVL1, 4 or 8 did not have any impact on HCV infection in Huh7.5 cells (Fig. 3c,d). Addition of an anti-IFNR antibody to supernatants from monocytes and pDCs stimulated with HCV RNA abolished their inhibitory effect on HCV infection, thus underlying the role of type I IFN in this mechanism (Fig. 3e,f). Of note, only supernatants from HCV RNA-stimulated monocytes and pDCs induced the phosphorylation of the tyrosine motif Y701 of STAT-1 in PBMCs, which also suggested that this inhibitory activity could be mediated by type I IFNs present in the supernatants of these cells (Supplementary Figure S1).

### HCV particles selectively stimulate macrophages via TLR7/8 but do not induce type I interferon

**Figure 4:**
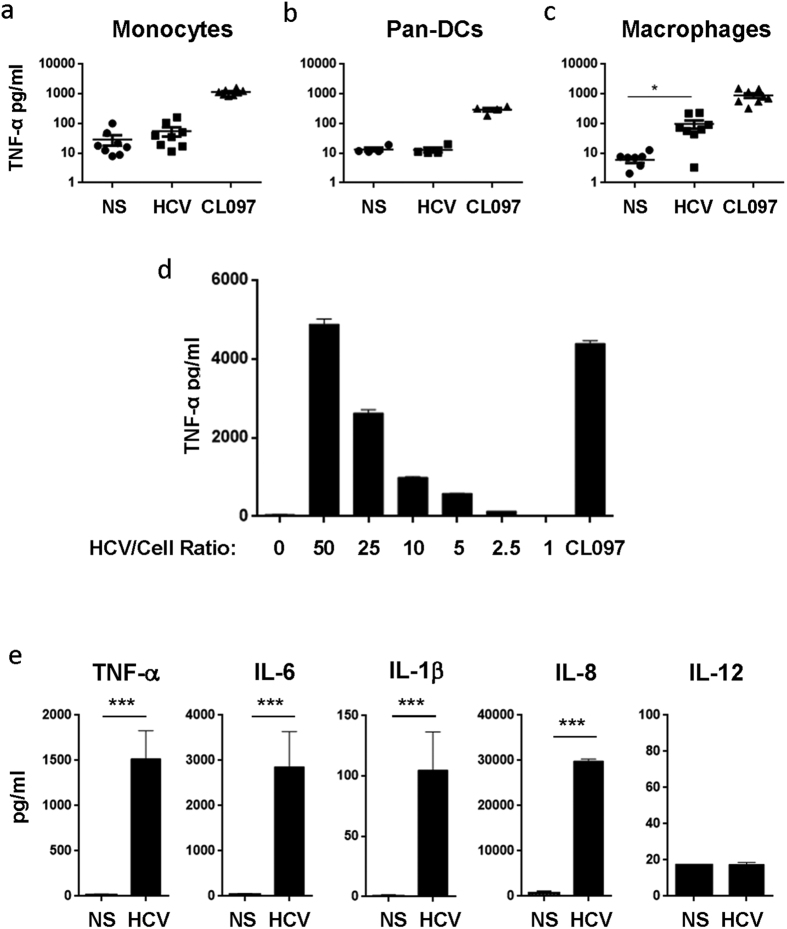
Macrophages but not DCs and monocytes are able to produce inflammatory cytokines upon exposure to HCV particles. **(a–c**) Monocytes (n = 8) and pan-DCs (n = 5) were isolated healthy donors by negative selection using magnetic beads (STEMCELL). Macrophages (n = 8) were generated from monocytes as described above. Monocytes (**a**), pan-DCs (**b**) and macrophages (**c**) (10^5^ cells/200 μl) were exposed to HCV particles (RNA copies/cells ratio of 20) or TLR7/8 agonist CL097 (1 μg/ml) for 24 h. (**d**) Macrophages (10^5^/200 μl) were stimulated with different ratios of HCV particles *vs*. cells (RNA copies/cells) for 24 h. **(a–d)** Levels of TNF-α in cell culture supernatant were measured by ELISA. (**e**) Levels of TNF-α, IL-6, IL-1β, IL-8, and IL-12p70 produced by macrophages upon stimulation of HCV particles were measured by CBA. Results represent mean ± SD values generated with cells from 5 different donors. Two tail paired student’s *t*-test *p*-values are indicated on the graphs. (*p < 0.05, **p < 0.01, ***p < 0.001).

Our results demonstrated that HCV RNA encodes sequences that stimulate APCs and induce anti-viral immune responses. We investigated the capacity of HCV viral particles to trigger such a response in different cellular subsets of APCs. Upon incubation with HCV particles for 24 hours, monocytes and DCs did not produce TNF-α. In contrast; these cells produced TNF-α upon incubation with the control CL097 ligand (Fig. 4a,b). Similar results were observed when total PBMCs were exposed to HCV particles (Supplementary Figure S2a). In contrast, both HCV particles, at the same number of RNA copies/ml/cells ratio that was used to stimulate the other APCs, and CL097 induced TNF-α production in macrophages (Fig. 4c). We further validated these observations on a large number of donors (n = 20) using a ratio of 20 HCV particles *per* cell. As expected HCV particles did not induce TNF-α production by monocytes, in contrast macrophages from 19 out of 20 donors responded to HCV stimulation as indicated by TNF-α production (average of 1,517 pg/ml, *p* < 0.0001; Supplementary Figure S2b).

We used LPS, CL097 and poly I:C as positive controls to monitor the responsiveness of these APCs in our assay. Both macrophages and monocytes responded to LPS (TLR4 ligand; an average of 2,200 pg/ml and 3,000 pg/ml, *p* = 0.0069 and 0.0072, respectively), CL097 (TLR7/8 ligand; an average of 3,000 pg/ml and 2,100 pg/ml, *p* < 0.0001 and *p* = 0.004, respectively) and poly I:C (TLR3 ligand; average of 900 pg/ml and 1,300 pg/ml, *p* = 0.0054 and 0.015, respectively). However, only macrophages but not monocytes produced TNF-α (2,850 pg/ml, *p* = 0.0032) upon stimulation with HCV particles (Supplementary Figure S2c,d), although both macrophages and monocytes were able to produce TNF-α in response to other RNA virus, such as Sendai virus (Supplementary Figure S2e,f). Moreover, HCV particles were able to induce TNF-α production by macrophages in a dose dependent manner, reaching 4,000 pg/ml of TNF-α when the ratio of HCV particles to cells were in the range of 50/1 (Fig. 4d). Furthermore, HCV particles stimulated the production of different inflammatory cytokines by macrophages such as IL-6 (2,800 pg/ml, *p* < 0.0001), IL-1β (110 pg/ml, *p* < 0.0001) and IL-8 (2,950 pg/ml, *p* < 0.0001; Fig. 4e). Interestingly, the production of the cytokine IL-12p70, which is crucial for the induction of CD4 T helper (Th1) 1 and CD8 T cell responses, was not induced by HCV particles in macrophages (Fig. 4e).

**Figure 5:**
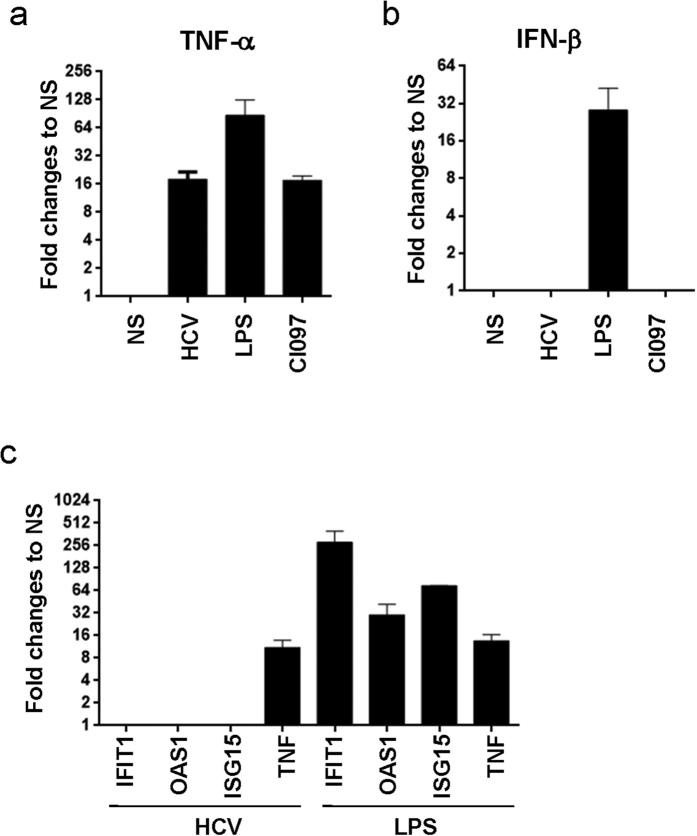
HCV particles fail to promote type-I IFN production in macrophages. **(a,b)** Monocyte-derived macrophages (10^5^/200 μl) were cultured in the presence or absence of HCV particles (RNA copies/cell ratio of 20), LPS (10 ng/ml), or CL097 (1 μg/ml) for 3 h. The expression of mRNA for TNF-α **(a)** and IFN-β1 **(b**) was measured by real-time PCR. (**c**) Macrophages (10^5^) were stimulated with HCV particles (RNA copies/cell ratio of 20) or LPS (1 ng/ml) for 16 h, then IFN-stimulated genes (IFIT1, OAS1, ISG15) and TNF-α mRNA levels were measured by real-time PCR. Results are shown as fold change to gene expression in stimulated cells *vs* non-stimulated cells (n = 3).

Macrophages were able to sense HCV particles, however cytokines produced upon this stimulation did not inhibit HCV replication (Fig. 3d) likely due to the absence of the type I interferon. To test this hypothesis, we monitored IFN-β and IFN-stimulated gene expression (IFIT1, OAS1, ISG15) in macrophages after 3 hrs incubation with HCV particles, LPS or CL097. Whereas TNF-α levels increased after stimulation with HCV particles, LPS or CL097 (Fig. 5a), the stimulation with the TLR7/8 agonist or HCV particles did not induce IFN-β expression by macrophages (Fig. 5b). To exclude the possibility that macrophages were unable to produce to produce IFN-β, we stimulated these cells with LPS. As shown in Fig. 5b, IFN-β was significantly up-regulated in LPS-stimulated macrophages (an average of 28-fold increase, values were 10, 20 and 56-fold increase in 3 independent donors) thus demonstrating the capacity of macrophages to produce IFN-β under other specific conditions. To further demonstrate the inability of HCV particles to induce the IFN pathway in macrophages, we measured the expression of IFN-stimulated genes (ISGs) at 16 hrs after stimulation with HCV particles or LPS. Although LPS induced the up-regulation of IFIT1 (an average of 285-fold increase, values were 90- 480- and 285-fold increase as observed in 3 donors), OAS1 (an average of 30-fold increase, values were 10- 30- and 50-fold increase as observed in 3 donors) and ISG15 (an average of 74.5-fold increase, values were 74- 74.5- and 75-fold increase as observed in 3 donors) in macrophages, the stimulation with HCV particles did not induce the up-regulation of these IFN-stimulated genes (Fig. 5c). Although stimulation of these macrophages with HCV particles or LPS led to the up-regulation of TNF-α expression in macrophages (11- and 13- fold increase respectively (Fig. 5c)). Interestingly, gene expression induced by HCV particles and TLR7/8 ligand CL097 has similar profiles, suggesting HCV likely triggers TLR7/8 in macrophages (Fig. 5a,b).

### HCV entry in macrophages is mediated by DC-SIGN

**Figure 6:**
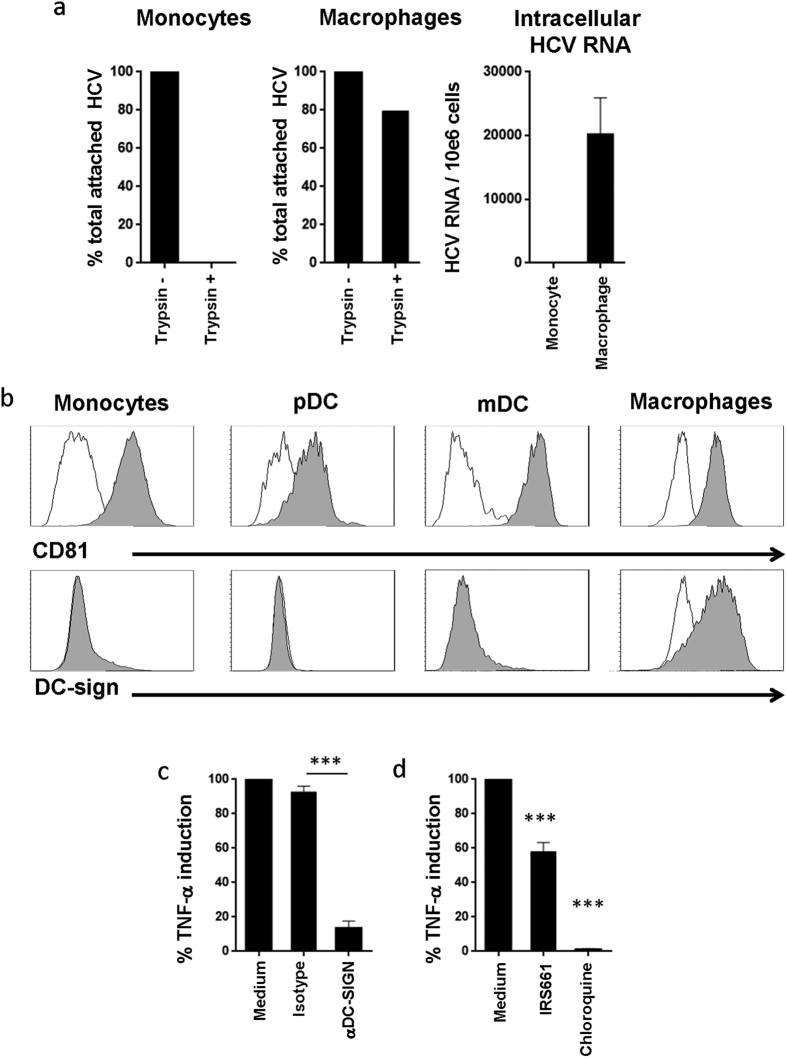
DC-SIGN and TLR7/8 are important for the uptake and recognition of HCV particles by macrophages. **(a)** Macrophages and monocytes (10^5^/200 μl) were incubated with HCV particles at 37 °C for 4 h, and then cells were washed and treated with trypsin at 37 °C for 5 mins to remove cell surface-attached viral particles. After extensive wash, total RNA was extracted and viral RNA was measured by real-time PCR. Viral RNA copies were normalized to GAPDH. Results were generated with cells from 3 different donors and represent the percentage of viral RNA associated to the cells untreated with trypsin. **(b)** PBMCs or Macrophages were stained with isotype control (open histograms), anti-CD81 or anti-DC-SIGN Abs (filled histograms). CD81 and DC-SIGN expression were measured on monocytes (CD14^+^), mDCs (Lin^−^CD14^−^HLA-DR^+^CD11c^+^CD123^−/dim^) and pDCs (Lin^−^CD14^−^HLA-DR^+^CD11c^−^CD123^+^) by flow cytometry using cells from 3 different donors. Macrophages were also stained with anti-CD81-PE or anti-DC-SIGN-PE Abs. (**c**) Macrophages were incubated with medium alone, isotype control Abs, or DC-SIGN blocking Abs (10 μg/ml) for 1 h, then cells were exposed to HCV particles for 24 h. Levels of TNF-α in cell culture supernatants were quantified by ELISA. Shown are percentages of TNF-α production relative to cells exposed to HCV particles alone. Results were generated with cells from 6 different donors. Two tail paired student’s *t*-T tests analysis-values are indicated on the graphs. (**d**) Macrophages were stimulated with HCV particles in the presence or absence of the TLR7/8 inhibitor IRS661 (10 μg/ml) or chloroquine (10 μM) for 24 h. TNF-α production was measured by ELISA. Results represent mean ± SD values generated with cells from 5 different individuals. Dunnett’s test p-values are indicated on the graphs (*p < 0.05, **p < 0.01, ***p < 0.0001).

We compared the capacity of macrophages and monocytes to uptake HCV particles to further investigate the mechanisms leading to their detection by these cells. To measure viral entry, monocytes or macrophages were incubated with HCV for 4 hrs followed by treatment with trypsin to remove the viral particles that remained attached to the cell surface. HCV particles bound to the surface of both macrophages and monocytes as we could detect viral RNA in both cell types by RT-PCR following the 4 hrs incubation period without trypsin treatment. Upon treatment with trypsin, viral RNA was not detected in monocytes, whereas it was still detected in macrophages (Fig. 6a).

We then investigated the implication of DC-SIGN, known to promote HCV endocytosis, in the specific capacity of macrophages to bind HCV particles. As a control we also monitored the role of CD81 in this process, as CD81 is known to be implicated in HCV entry. Our results showed that whereas macrophages, monocytes, pDCs and mDCs all expressed CD81, DC-SIGN was exclusively expressed by macrophages (Fig. 6b). To assess the potential role of DC-SIGN in the recognition of HCV particles by macrophages, we used a DC-SIGN neutralizing antibody that blocks the interaction of DC-SIGN with HCV envelop protein^23^. Macrophages were incubated with HCV particles in the presence or absence of the anti-DC-SIGN blocking antibody or with its matched isotype control. TNF-α production by macrophages treated with anti-DC-SIGN was significantly decreased (an average of 90% inhibition, *p* < 0.0001; Fig. 6c), which indicates that DC-SIGN is involved in the uptake of HCV by macrophages. Interestingly, we observed a much higher expression of DC-SIGN on monocytes-derived DCs (MDDCs) as compared to macrophages (an average MFI 40,774 and 4,579 respectively, *p* = 0.0043; Supplementary Figure S3a). However, these MDDCs did not respond to HCV particles as monitored by TNF-α production. In contrast, MDDCs were able to respond to stimulation by CL097 and Sendai virus (Supplementary Figure S3b). Our data showed that DC-SIGN is involved in HCV recognition by macrophages. However, for the stimulation to take place other downstream mechanisms are likely required as MDDCs express high levels of DC-SIGN but are not triggered by HCV particles.

As we described above, TLR7/8 are likely involved in sensing HCV particles. To assess this hypothesis, we tested the capacity of IRS661 and chloroquine, a specific inhibitor of endosomes acidification, to inhibit the stimulation of macrophages by HCV particles. IRS661 inhibited 50% of TNF-α production induced by viral particles (Fig. 6d, *p* < 0.0001), indicating an important role for TLR7/8 in detecting HCV particles by macrophages. Interestingly, chloroquine completely abolished TNF-α production by macrophages exposed to HCV particles (Fig. 6d, *p* < 0.0001), which indicates that this process was mediated by endosomal TLRs.

Together, our data showed that recognition of HCV RNA by monocytes and pDCs mediated anti-HCV functions in an IFN-dependent fashion. However, HCV complete particles, although they were able to stimulate macrophages but not other APCs, failed to induce the production of IFN-β by macrophages; therefore, they were compromised in their antiviral activity.

## Discussion

In the current study we identified novel HCV-encoded RNA sequences that can elicit TLR7/8-mediated innate immune responses. We show here that the presence of GU-rich sequences in HCV ssRNA are essential but not sufficient to stimulate immune responses as 6 out of the 9 GU-rich ssRNA sequences identified in our study did not induce detectable responses. This suggests that other parameters might also participate in determining the capacity of viral ssRNA to trigger TLR7/8. Moreover, other sequences with the capacity to stimulate TLR7/8 might also exist in HCV genome. HCV-encoded RNA sequences that we identified here stimulated inflammatory cytokines and chemokines, as well as type I IFN-dependent anti-HCV responses by primary APCs, particularly monocytes, mDCs and pDCs. These responses could not be initiated by HCV particles as monocytes and DCs were defective in their capacity to sense HCV particles. Only macrophages were able to sense HCV particles, however, this induced a pro-inflammatory environment, which is prevalent in HCV infected subjects as it was previously shown to be associated to disease progression^24^,^25^. Importantly, HCV particles failed to induce the production of type I IFN, thus allowing HCV to escape the intrinsic antiviral innate immune response.

The lack of DC and monocyte activation by HCV is in line with earlier reports showing a deficiency in virus uptake by these cells^26^,^27^. Our results suggest that this is likely a result of a blockade at the entry step, which might be due to the lack of DC-SIGN expression in primary DCs and monocytes. DC-SIGN plays a key role in the uptake of HCV as previously shown *in vitro* by monocyte-derived DCs^23^,^28^ and further confirmed by our current data; we demonstrated that macrophage activation by HCV particles is DC-SIGN-dependent. Although, DC-SIGN is not expressed by peripheral DCs^29^,^30^, it is expressed on dermal DCs, ‘monocytoid’ DCs in the lymph nodes and macrophages in the liver^31^,^32^,^33^,^34^,^35^, which suggests that these cells could sense HCV in its target tissue. DC-SIGN expression does not warrant optimal responses upon recognition of HCV since we show that monocyte-derived DCs, although expressing high levels of DC-SIGN, did not respond to HCV particles. This lack of response was specific to HCV as these same cells strongly responded to Sendai virus, which is another RNA virus. In line with this hypothesis, earlier reports have shown that immature DCs can readily uptake HCV particles through DC-SIGN-mediated internalization into a non-lysosomal compartment, where virus escapes the detection by TLRs^28^,^36^.

The lack of detection of HCV particles did not apply to all innate APCs as we showed here that macrophages could indeed recognize HCV particles through a DC-SIGN-dependent interaction, which leads to the triggering of TLR7/8 signaling, as confirmed by the use of TLR antagonists, and the production of pro-inflammatory cytokines. Whether the differentiation of monocytes into macrophages *in vitro* have impacted their activation profile, and consequently enhanced their potential to detect HCV particles remains unknown and deserves further investigations. However, the *in vitro* differentiation of monocytes to MDDCs did not render them sensitive to the stimulation with HCV particles, which suggests that the ability to detect HCV particles required monocytes differentiation into macrophages.

Moreover, type I IFN and IFN-stimulated genes, i.e. IFIT1, OAS1, and ISG15 gene expression signature, which are key mediators of viral inhibition^37^,^38^,^39^,^40^, were not induced upon stimulation of macrophages by HCV particles. The biased IFN signal in response to HCV in macrophages is not likely specific to HCV but rather reflects an altered type I IFN response upon triggering of TLR7/8 in these cells. For instance, activation of macrophages with chemical ligands of TLR7/8, i.e. CL097, did not induce any significant interferon production by these cells. However, upon activation with LPS, which triggers TLR4, the same macrophages produced IFN-β. Those results suggest that activation of TLR7/8 pathway in macrophages does not lead to type I IFN production.

One of these potential mechanisms that could explain the lack of type I interferons production by these cells could involve NLRX-1, an inhibitor of mitochondrial antiviral immunity, which is known to prevent the induction of antiviral response in macrophages^41^,^42^. Moreover, alternatively activated STAT1 has been shown to negatively regulate TLR7/8 signaling in macrophages, especially prevent type I IFN production^43^. The antagonist effects of IL-1β may also contribute in the absence of IFN production in macrophages as IL-1β inhibits IFN-β synthesis^44^. IL-1βinhibits type I IFN responses by directly promoting IFN receptor turnover^45^ and antagonize STAT1 activity^46^. Moreover, the absence of type I IFN production by macrophages upon stimulation with HCV particles might be due to the fact that TGF-β and IL-10, which are produced by macrophages themselves, have the capacity to negatively regulate TLRs-induced type I IFN production^47^,^48^; this possibility remains to be investigated in future studies.

HCV particles selectively activated macrophages to induce pro-inflammatory cytokines, such as IL-1β, TNF-α and IL-8, which have been shown to be up-regulated in chronically HCV-infected individuals and correlate with HCV-driven fibrogenesis^10^,^25^,^49^,^50^,^51^. This may further suggest that activation of macrophages by HCV can potentially contribute to disease progression in infected patients by this virus. In line with this hypothesis, resident hepatic macrophages, Kupffer cells (KCs), in HCV-infected subjects were shown to be the primary cellular source of liver inflammation as they produce significantly high levels of IL-1β upon exposure to HCV^10^. The production of IL-1β, also observed in our experiments, indicates the capacity of HCV particles to activate the inflammasome in macrophages^10^. While macrophages sensed HCV particles, this did not trigger IL-12p70 production and consequently this detection might not be sufficient to prime potent Th1 and CD8 T cell responses. Hence our results indicate that macrophage sensing of HCV particles triggers a pro-inflammatory response that does not include the pathways and cytokines required for the development of a protective adaptive cellular immune response. Liver macrophages are stimulated during chronic HCV infection, however their profile suggests that they do not support the induction of proper T cell responses^52^. The capacity of resident and infiltrating macrophages in the liver to detect HCV particles and produce anti-viral type-I IFNs remains to be investigated as these cells might have some differences compared to monocytes-derived macrophages. Of note, HCV core protein (HCVc) has been found to be able to block TLR3-mediated secretion of IFN-α, IFN-β by human KC^53^.

In conclusion, in this study we have shown that innate immune responses mediated by HCV particles were limited to macrophages as neither monocytes, mDCs nor pDCs have the potential to detect HCV viral particles. However, activation of macrophages by HCV is not optimal and does not trigger anti-viral responses but rather induces a pro-inflammatory response. Thus, the development of an anti-HCV vaccine should consider strategies that lead to a proper stimulation of TLR7/8 in monocytes, mDCs and pDCs in order to improve the quality of the immune response against HCV^54^,^55^. Our results thus pave the way for a better understanding of the essential innate immune responses to HCV, which are key elements for the development of an effective anti-HCV vaccine.

## Materials and Methods

### Cell lines and reagents

Hepatoma Huh7.5 cell line (provided by Dr. Charles Rice, Rockefeller University) and Huh7.5–20 producing JFH1 HCV (provided by Dr. T. Jake Liang, National Institutes of Health) were cultured in DMEM-based complete growth medium. The 293HEK cell line expressing TLR3, TLR7 or TLR8, and the plasmid pNiFty2-Luc were obtained from Invivogen (USA). The pNiFty2-Luc plasmid contains a reporter gene expression cassette that allows firefly luciferase production under the control of NF-κB. Nine sequences within the HCV genome containing around 65% GU were selected and synthesized (Integrated DNA Technologies, Canada). TLR7/8 agonist RNA40, CL097 and TLR3 agonist poly I:C were obtained from Invivogen. DC-SIGN blocking antibody and matched isotype control were obtained from R&D Systems.

### Antibodies used for flow cytometry

The following Abs were used for flow cytometry: CD80-FITC, CD86-PE, CD14-Pacific blue, Linage-Alexa700 (CD3, CD19, CD16), HLA-DR-APC-cy7, CD11c-APC, CD123-FITC, CD81-PE (BD Bioscience, USA), DC-SIGN-PE (R&D Systems, USA). A viability dye (Molecular Probes® LIVE/DEAD® Fixable Dead Cell Stain Kits, Invitrogen) was used to exclude dead cells. Cells were stained and analyzed by FACS using the BD LSRII cytometer and FlowJo software.

### Cell sorting and differentiation

Human peripheral blood mononuclear cells (PBMCs) were isolated from leukapheres using ficoll density centrifugation. Enrichment kits for monocytes, plasmacytoid dendritic cells (pDCs) and panDC separation were obtained from STEMCELL Technologies Inc. Myeloid DCs (mDCs) were isolated from PBMCs using the CD1c^+^ Dendritic Cell Isolation Kit (Miltenyi Biotec, USA). Typically, the purity of cell fractions was >98% as determined by staining with anti-CD14-PB Abs for monocytes, and with lineage-Alexa700 (CD3, CD14, CD19, CD16), HLA-DR-APC-Cy7, CD11c-APC and CD123-PE Abs for DCs. To prepare macrophages, monocytes were cultured in the X-VIVO^TM^ 15 medium (lonzabio, USA) for 6 days in the presence of GM-CSF (50 ng/ml; R&D Systems, USA). To obtain monocyte-derived DCs (MDDCs), monocytes were cultured for 6 days in the X-VIVO^TM^ 15 medium supplemented with GM-CSF (50 ng/ml) and IL-4 (20 ng/ml). Informed consent was obtained from all the donors and the project was approved by the Institutional Review Boards (IRB) of the Centre Hospitalier de l′Université de Montréal Research Center, Vaccine & Gene Therapy Institute of Florida.

### Cytokine quantification

TNF-α production was measured using the Human TNF alpha ELISA Ready-SET kit (eBioscience, USA). In parallel, cytokines (IL-1β, IL-6, IL-10, IL-12p70 and TNF-α) and chemokines (CXCL9, CXCL10) were measured by Cytometric Bead Array (CBA) using the Human Inflammatory Cytokine Kit and Human Chemokine Kit (BD Biosciences, USA). Type I IFN was measured using the Human Interferon alpha ELISA kit and the Human Interferon Beta ELISA Kit (PBL Interferon Source, USA).

### Real-time RT-PCR

RNA was extracted using RNeasy Mini Kit (QIAGEN, USA). The expression of mRNA for TNF-α and IFN-β, together with the mRNA for the IFN-stimulated genesIFIT1, OAS1 and IAS15, was measured using predesigned primers and probe sets (Roche-Applied-Science) and the LightCycler® 480 Probes Master(ROCHE, USA). Real-time RT-PCR (Qiagen) was carried out in a LightCycler 480 II (Roche) according to manufacturer’s recommendations. The expression of each gene in different samples was normalized relative to GAPDH mRNA levels.

### HCV production and quantification

The Huh7.5–20 cells stably producing HCV particles were cultured in DMEM-based complete growth medium for 4 days. The cell culture supernatant was harvested and filtered through a 0.45 μm filter to remove cell debris (Sarstedt, Germany). To concentrate the virus, supernatant containing HCV particles was passed through an Amicon ultra-15 centrifugal filter-100 (Millipore, USA). Filters were sterilized with 70% alcohol and rinsed with PBS before use. Concentrated viral aliquots were stored at −80 °C for further use. The viral titer was quantified by real-time RT-PCR. Briefly, RNA was extracted from cell culture supernatant using the QIAamp Viral RNA Mini Kit (QIAGEN, USA). Real time PCR was performed as described above using the specific forward primer 5′-CGGGAGAGCCATAGTGG-3′, reverse primer 5′-AGTACCACAAGGCCTTT-3′, and the probe 5′-/56-FAM/CTGCGGAACCGGTGAGTACAC/3IABlkFC (IDT, USA).

### Study approval

This study was approved by the Institutional Review Boards (IRB) of the Centre Hospitalier de l′Université de Montréal Research Center, Vaccine & Gene Therapy Institute of Florida and at all participating sites’ IRBs. All experiments were performed in accordance with the guidelines and regulations approved by the ethic committees from CRCHUM, VGTIFL and all IRBs. Study participants provided written informed consent for the use of their plasma and cells for the current research investigation.

### Statistical analysis

Statistical analyses were performed using the GraphPad Prism 5. Details are included in Figure legends. Actual *p*-value is showed in the main text when it is available.

## Additional Information

**How to cite this article**: Zhang, Y. *et al*. HCV RNA Activates APCs via TLR7/TLR8 While Virus Selectively Stimulates Macrophages Without Inducing Antiviral Responses. *Sci. Rep*. **6**, 29447; doi: 10.1038/srep29447 (2016).

## Supplementary Material

Supplementary Information

## Figures and Tables

**Table 1:** Cytokines and chemokines production by monocytes, mDCs and pDCs.

Cell type	Ligand	TNF-a	IL-6	IL-1β	IL-10	IL-12	CXCL9	CXCL10
monocyte	NS	45	61	124	34	41	68	680
	cl097	8251	11884	5392	153	93	183	970
	DOTAP	54	112	133	34	39	73	817
	HCVL1	9344	12383	4927	75	157	408	1189
	HCVL1mut	48	961	196	41	37	66	681
	HCVL4	7252	12267	2803	205	66	360	1092
	HCVL4mut	53	205	131	65	44	62	679
	HCVL8	12483	12704	5540	223	176	355	1135
	HCVL8mut	276	3780	333	57	39	89	877
mDC	NS	287	4266	382	84	48	96	716
	cl097	9397	10443	6335	188	400	1122	877
	DOTAP	120	317	282	51	37	111	726
	HCVL1	3638	6354	3355	153	179	1427	1587
	HCVL1mut	59	391	302	55	37	92	680
	HCVL4	4060	8545	2115	80	200	1527	1483
	HCVL4mut	41	352	215	51	39	128	760
	HCVL8	6383	8528	3391	138	329	1432	1666
	HCVL8mut	79	433	261	53	43	91	743
pDC	NS	37	564	129	38	37	1087	1177
	cl097	1693	2257	224	60	74	13623	5653
	DOTAP	50	43	110	30	42	304	1899
	HCVL1	2155	2047	202	56	61	8349	6370
	HCVL1mut	40	47	109	33	41	362	2473
	HCVL4	1883	2370	183	68	48	9793	5862
	HCVL4mut	45	52	110	33	45	786	1662
	HCVL8	5476	2350	170	59	87	9309	6975
	HCVL8mut	58	55	109	35	38	328	2051

Monocytes, mDCs and pDCs (10^5^/200 μl) were isolated from 3 healthy donors by negative selection using magnetic beads and stimulated with the indicated HCV ssRNA sequences (7.5 μg/ml). The supernatants were harvested at 24 h post-stimulation. Levels of cytokines TNF-α, IL-6, IL-1β, IL-10, IL-12p70, and chemokines CXCL9, CXCL10 were measured in the cell culture supernatant by CBA. The U to A mutated forms of HCVL1, HCVL4 and HCVL8 were used as negative controls. Values represent mean of results generated with cells from 3 different donors.
