# Supplementary material for: HCV RNA Activates APCs via TLR7/TLR8 While Virus Selectively Stimulates Macrophages Without Inducing Antiviral Responses
Source: Sci Rep. 2016 Jul 7;6:29447. doi: 10.1038/srep29447 (PMC4935957; doi:10.1038/srep29447)
Supplement: Supplementary Information [file srep29447-s1.pdf]

HCV RNA Activates APCs via TLR7/TLR8 While Virus Selectively Stimulates Macrophages Without Inducing Antiviral Responses

Yuwei Zhang[1-3], Mohamed El-Far[1,2], Franck P. Dupuy[1-3,5], Mohamed Salah Abdel-Hakeem[1,4], Zhong He[1-3], Francesco Andrea Procopio[1-3], Yu Shi[1-3], Elias K. Haddad[1-3], Petronela Ancuta[1,2], Rafick-Pierre Sekaly[1-3, 6], Elias A. Said[*1, 2, 7]

Supplementary information

Supplementary Figure Legends

Supplementary Figure S1: STAT1 phosphorylation reveals type I IFN activity in supernatants from HCV ssRNA-exposed monocytes and pDCs but not mDCs. Monocytes, mDCs, and pDCs were isolated from 3 healthy donors by negative selection using magnetic beads. Cells were stimulated with the newly HCV ssRNA sequence HCVL1 (7.5 µg/ml). Cell-free supernatants (SN) were harvested at 24 h post-stimulation. PBMCs (10^6/ml) were incubated for 15 min. with SN from monocytes, mDCs, or pDCs that were exposed to medium only (blue), DOTAP alone (Red), or HCV ssRNA-DOTAP complex (Green). Cells were then fixed and stained with anti-phosphorylated STAT1 (Y701) Ab according to the manufacturer's Phosflow protocol (BD Biosciences). Cells were analyzed by flow cytometry for the intracellular expression of phosphorylated STAT1.

Supplementary Figure S2: Macrophages but not monocytes produce TNF-α in response HCV particles. (a) PBMCs (10^5/200µl) from 3 different donors were incubated with HCV particles (virus/cell ratio of 100), DOTAP alone or HCV RNA-DOTAP complex for 24 h. **(b)** Macrophages or monocytes (10^5/200µl) were stimulated with HCV particles at a virus/cell ratio of 20 for 24h. TNF-α production was measured by ELISA. Results were generated with cells from twenty different healthy donors. Macrophages **(c)** and monocytes **(d)** were stimulated with HCV particles, medium, CL097 (1µg/ml), polyI:C (30µg/ml) or LPS (1ng/ml) for 24h. Macrophages **(e)** and monocytes **(f)** were stimulated with Sendai virus (200 hemagglutinating units/ml) or CL097 for 24h. Supernatants were harvest at 24 h post-stimulation and TNF-α production was measured by ELISA (n=3).

Supplementary Figure S3: Both monocyte-derived macrophages and DCs express DC-SIGN but only macrophages are stimulated by HCV particles. Monocytes were cultured in the presence

of GM-CSF or GM-CSF/IL-4 for 6 days to generate macrophages and MDDCs, respectively. (a-b) DC-SIGN expression on macrophages and DCs was measured by flow cytometry. Shown are representative results generated with cells from one donor (a) and statistical analysis of DC-SIGN MFI expression on cells from 3 different donors (b, c) Macrophages and MDDCs (10^5/200µl) were stimulated with HCV particles at a virus/cell ratio of 20, or with CL097 (1µg/ml) or Sendai virus (200 hemagglutinating units/ml) for 24h. TNF-α production was measured by ELISA. Results represent mean $\pm$ SD of values generated with cells from three different donors.

Supplementary Table S1: Novel GU-rich sequences in the HCV genome. GU-rich ssRNA sequences were analyzed within HCV genome based on the sequence of JFH-1 strain. Nine sequences (HCVL1 to HCVL9) that contain around 65% G/U or more were selected.

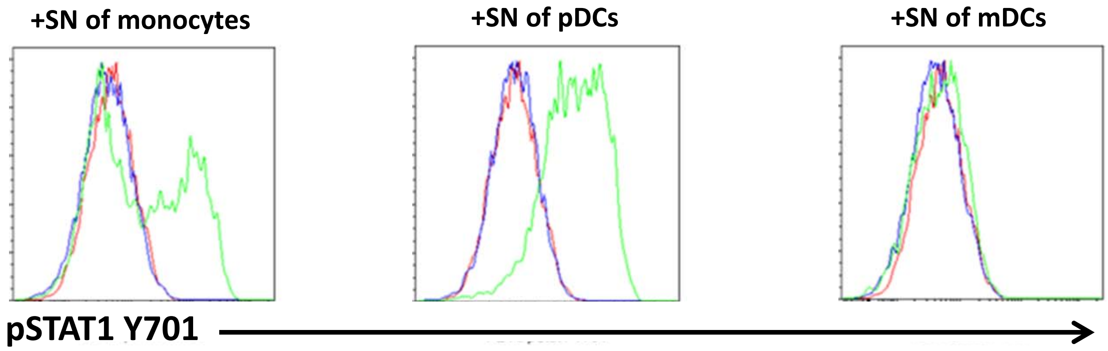

Zhang et al., Supplemental Figure S1

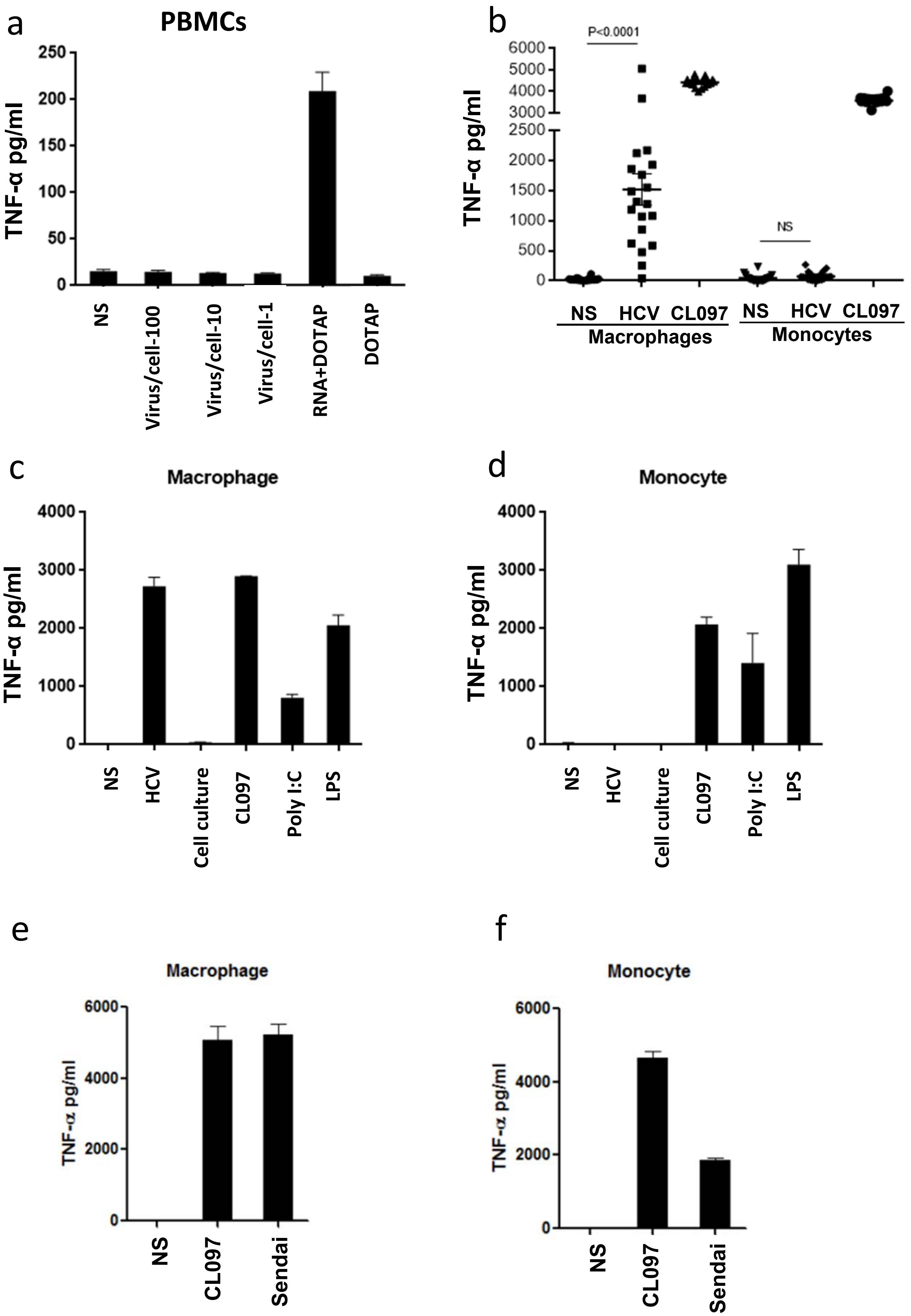

Zhang et al., Supplemental Figure S2

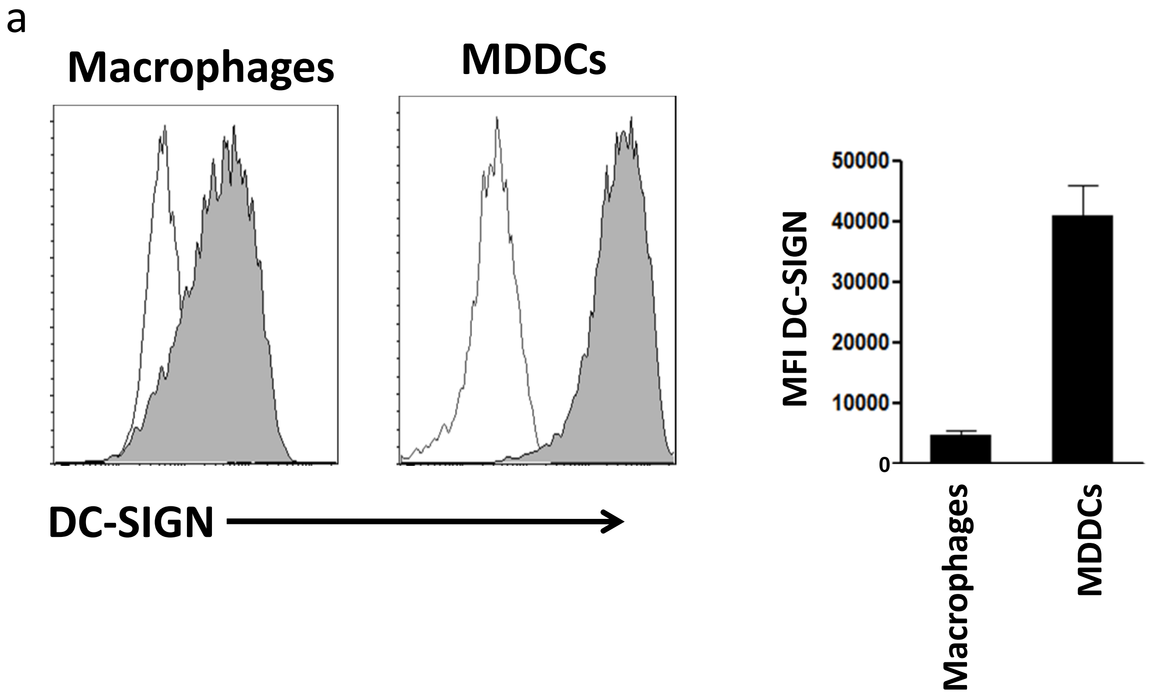

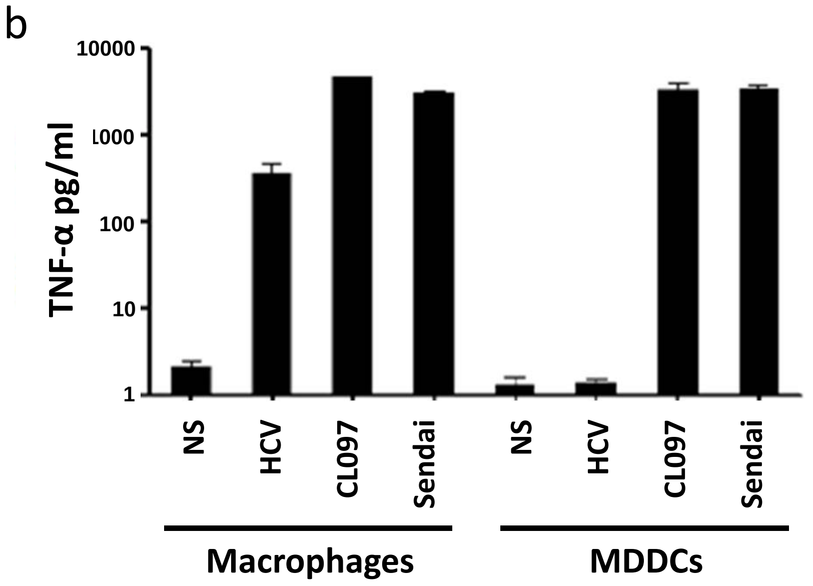

Zhang et al., Supplemental Figure S3

Supplemental Table S1

ID	RNA sequences	Regions
**HCVL1**	GUAGUGUUGGGUCGCGAAAGGCCUUGUGGU	5'UTR
**HCVL2**	GUCAAGUUCCCGGGUGGCGGUCAGAUCGUUGGUGGAGUUUACUUGUUG	Core
**HCVL3**	UGUGCGGGUCUGUCUUUCUUGUUGGUCAACUGUUUACCUU	E1
**HCVL4**	UUGUGGACGUGCAGUACUUGUACGGGGUAGGGU	E2
**HCVL5**	GUCUUGUGUCCUUCCUCGUGUUCUUCUGCUUUGCGUGGUAUCUGAAGG GUAGGUGGGU	p7
**HCVL6**	GUCUUGUGUCCUUCCUCGUGUUCUUCUGCUUUGCGUGGU	p7
**HCVL7**	GUGGCCGCGUCGUGUGGCGGCGUUGUUCUUGUCGGGUU	NS2
**HCVL8**	GUUGUCGUCGUGUCGACCGA	NS3
**HCVL9**	GUGUGUGGCGACGACUUAGUCGUUAUCUGUGAAAGUG	NS5B
